# Immunopathogenesis of primary biliary cirrhosis: an old wives' tale

**DOI:** 10.1186/1742-4933-8-12

**Published:** 2011-12-02

**Authors:** Daniel S Smyk, Eirini I Rigopoulou, Ana Lleo, Robin D Abeles, Athanasios Mavropoulos, Charalambos Billinis, Pietro Invernizzi, Dimitrios P Bogdanos

**Affiliations:** 1 Institute of Liver Studies, King's College London School of Medicine at King's College Hospital and Kings College Hospital NHS Trust Foundation, London, SE5 9RS UK; 2 Department of Medicine, University of Thessaly Medical School, Viopolis, 41110, Larissa, Greece; 3 Institute of Biomedical Research and Technology (BIOMED), CERETETH, 41222, Larissa, Greece; 4 Center for Autoimmune Liver Diseases, Division of Internal Medicine, IRCCS Istituto Clinico Humanitas, Rozzano 20089, Italy; 5 Department of Translational Medicine, Università degli Studi di Milano, Rozzano, Italy; 6 Faculty of Veterinary Medicine, University of Thessaly, Karditsa, Greece; 7 Division of Rheumatology, Allergy and Clinical Immunology, University of California at Davis, Davis, CA 95616, USA

**Keywords:** ageing, apoptosis, autoantibody, autoimmunity, infection

## Abstract

Primary biliary cirrhosis (PBC) is a cholestatic liver disease characterised by the autoimmune destruction of the small intrahepatic bile ducts. The disease has an unpredictable clinical course, but may progress to fibrosis and cirrhosis. Although medical treatment with urseodeoxycholic acid is largely successful, some patients may progress to liver failure requiring liver transplantation. PBC is characterised by the presence of disease specific anti-mitochondrial (AMA) antibodies, which are pathognomonic for PBC development. The disease demonstrates an overwhelming female preponderance and virtually all women with PBC present in middle age. The reasons for this are unknown; however several environmental and immunological factors may be involved. As the immune systems ages, it become less self tolerant, and mounts a weaker response to pathogens, possibly leading to cross reactivity or molecular mimicry. Some individuals display immunological changes which encourage the development of autoimmune disease. Risk factors implicated in PBC include recurrent urinary tract infection in females, as well as an increased prevalence of reproductive complications. These risk factors may work in concert with and possibly even accelerate, immune system ageing, contributing to PBC development. This review will examine the changes that occur in the immune system with ageing, paying particular attention to those changes which contribute to the development of autoimmune disease with increasing age. The review also discusses risk factors which may account for the increased female predominance of PBC, such as recurrent UTI and oestrogens.

## Introduction

### Primary Biliary Cirrhosis: a disease of Women

Primary biliary cirrhosis (PBC) is a chronic cholestatic liver disease of autoimmune origin, characterised by inflammatory destruction of the small intrahepatic bile ducts, and fibrosis which can progress to cirrhosis and subsequent liver failure [1-3]. Symptoms include fatigue, insomnia, pruritus, Sicca symptoms, and arthralgias. More severe symptoms relate to portal hypertension and hepatic decompensation [1-3]. PBC shows a high female preponderance, mainly affecting middle aged women [1-4]. The female to male ratio ranges from 9:1 to 20:1, which is relatively higher than other autoimmune diseases [2]. Diagnosis requires the presence of at least two out of three of the following: antimitochondrial antibodies (AMA) in serum, biochemical markers of cholestasis, and characteristic histopathological features [2,5-7]. AMA and disease-specific anti-nuclear antibodies (ANA) are usually present at high titres [2,5,6,8]. Many 'non-autoimmune' liver disorders such as viral hepatitides and acute liver failure are characterised by the presence of autoantibodies, however, when AMA are present they are often suggestive of co-existing PBC [6,9-18]. In these non-autoimmune driven liver pathologies, it is not clear whether AMA or other autoantibodies just indicate immune dysregulation or are pathologically relevant [19-25].

AMA are present in 95% of PBC patients, with a specificity of nearly 100% [6,7,20,21,26,27], therefore true AMA-negative PBC can exist but is extremely rare [28]. AMA is primarily of the IgG isotype, however, IgA and IgM are also detected [5]. As the presence of AMA is so strongly associated with PBC, it is generally viewed that asymptomatic patients who are AMA positive will eventually develop PBC [29,30]. Patients with PBC exhibit a multi-lineage response to the E2 subunit of the pyruvate dehydrogenase complex (PDC-E2), which involves AMA as well as autoreactive CD4 and CD8 responses [6,31-33]. Histological features typical for PBC include: destruction of biliary epithelial cells (BEC), loss of small bile ducts (ductopaenia) with portal inflammatory cell infiltration; and occasional granuloma formation [1-3,34]. The portal inflammation is comprised largely of CD4, CD8, and B cells, as characterised by immunohistochemistry [2,33,35].

### PBC: A disease of the old

PBC is overwhelmingly a disease of middle-aged and older females, and is virtually absent in the paediatric or adolescent population, although several case reports indicate the development of PBC in children [23,36-41]. On closer analysis, these cases rarely represent true (or confirmed) PBC, with several of these cases representing AMA positivity in the absence of clinical evidence of disease [41], or AMA positivity in the presence of liver disease other than PBC [23,38]. Other cases represent PBC-like disease, which result from genetic mutations in genes controlling immunological regulation [36,40]. For example, Aoki and colleague report the case of a six month old male with abnormal liver biochemistry, and AMA positivity [36]. Histology in that case was not characteristic of PBC and the child was eventually diagnosed with IL-2-receptor- alpha deficiency, which resulted in defects of T regulatory cells [36]. This is similar to another case report by Tsuda and colleagues, who noted AMA positivity in an 11 year old with IPEX syndrome, a congenital disorder of immune regulation [40]. Although AMA positivity is viewed as being predictive of eventual PBC development in adults, it is unclear as to whether this applies in the paediatric population.

Only two case reports indicate genuine cases of paediatric PBC. Melegh et al [39] notes the case of a six year old female with AMA positivity and histological features of PBC, which progressed to fibrosis. The AMA reactivity in that case was atypical to most PBC cases, in that specificity was to PDC-E3 related subunit as well as anti-citrate synthase [39]. Dahlan and colleagues report PBC in two children, one at eleven years, and the other at 15 years [37]. Follow-up of the eleven year old showed that she progressed from stage II PBC at 16 years, to stage IV PBC at 21 years of age, when she was transplanted [37]. Interestingly, her 30 year old mother also had PBC. In summary, true paediatric PBC is exceedingly rare, and likely represents an aetiopathogenesis which is highly atypical compared to most cases of PBC.

### Why is PBC a disease of older women?

Although the aetiopathogenesis of autoimmune gastrointestinal and liver diseases remains poorly understood, it is believed that genetic susceptibility, immune dysregulation and exposure to environmental triggers are responsible for antigen-specific immunological breakdown that lead to the development of the disease [22,42-51]. The exact mechanisms by which environmental agents can trigger immunological breakdown are not well defined, but in most cases self/non-self mimicry has been considered to be instrumental [52-60]. Various environmental triggers have been implicated in the development of PBC, including xenobiotics, infectious agents, and hormones [49,52,61-71], but why PBC primarily affects women in their fifth or sixth decade is still unanswered.

This review will address the current evidence surrounding the high female preponderance of PBC within a particular age group. Current evidence relating to the ageing immune system suggests an increased risk of developing autoimmune disease in later life (Table 1). In addition, risk factors for PBC, such as recurrent urinary tract infections (UTI), reproductive history and oestrogen deficiency, appear to increase the susceptibility towards disease development in females (Table 2).

**Table 1:** Features of immunological ageing seen in the cellular pathology of primary biliary cirrhosis (PBC).

Ageing Feature	Presence in PBC
Decreased regulatory T cell (Treg) response	- Increased CD127 and decreased CD39 on CD8+ T cells
	-CD8+CD28- cells fail to display a regulatory response when incubated with IL-10
Decreased telomere length	-Quantitative fluorescence in situ hybridisation has shown decreased telomere length in biliary epithelial cells (BEC) of primary biliary cirrhosis (PBC) patients
DNA damage	- gammaH2AX-DNA-damage-foci detected in BEC
Increased apoptosis	-Apoptotic marker Bcl-2 in BEC (may increase PDC-E2 exposure)
	-Increased apoptotic marker CD95 (Fas) on BEC
	-Unmodified PDC-E2 found in apoptotic blebs (apotopes) in BEC
Increased cellular senescence	-Increased expression of senescence markers p16 and p21 in BEC
	-Decreased Ki67 expression in BEC, indicating decreased cellular proliferation
	-Decreased Bmi-1 (senescence regulator) expression in BEC
Increased autophagy	-Increased LC3 expression in BEC, which correlated with increased autophagy

Several features characterise an aged immune system, including changes in T cell response, decreased telomere length and DNA damage, as well as apoptosis and cellular senescence. These features have also been demonstrated in liver tissues from patients with PBC. These findings may, in part, explain why PBC patients often present in middle age, compared to other autoimmune diseases. Please see relevant sections in the text for more detailed descriptions.

**Table 2:** PBC risk factors associated with female sex and ageing

Female Risk Factor	Significance
Recurrent urinary tract infections	Molecular mimicry and cross reactivity with bacterial (such as E. coli) peptides and PDC-E2 epitopes
Oestrogen deficiency	Increased propensity towards ductopaenia
Increased incidence of vaginal infections	Alterations in vaginal flora increase the risk of developing urinary tract infections, which appear to play a role in PBC
**Ageing Risk Factors**	**Significance**
T cell membrane rigidity and decreased cell membrane fluidity	Alterations in T cell receptor signalling
Decreased CD28 expression (especially in CD8+ cells)	Reduced regulatory T cell function and reduced immune response to infections
Phenotypical alterations in CD8+ cells (such as increased CD127, and decreased CD39)	Decreased T regulatory cell function
Increased telomere shortening from oxidative stress an ongoing inflammation	Increased cellular senescence, autophagy and apoptosis
Increased senescence and autophagy	May contribute to exposure of self antigens to the immune system, as well as contributing to a pro-fibrotic and pro-inflammatory environment
Increased apoptosis	Formation of apoptotic blebs (apotopes) increase exposure of mitochondrial antigens to the immune system

Several risk factors related to female sex and increased age appear to be significant in the pathogenesis of primary biliary cirrhosis (PBC). Risks associated with females include recurrent urinary tract infections, oestrogen deficiency, and increased incidence of vaginal infections. Age related changes (which may increase risk for PBC development) include alterations in regulatory T cell function, increased senescence and autophagy, as well as apoptosis.

### The ageing immune system and impaired regulatory mechanisms

Out of all autoimmune diseases, PBC has one of the latest ages of onset, followed by systemic sclerosis and Sjogren's syndrome [72]. Intriguingly, Sjogren's syndrome (and to a lesser extent systemic sclerosis) co-exist in a significant proportion of patients with PBC. This raises the question as to what role 'immunosenescence', the term used to describe the ageing of the immune system, plays a role in the pathogenesis of these diseases. Immunosenescence begins in approximately the fifth or sixth decade of life, and is characterised by an age dependant, progressive decline in immunological functioning [72,73]. Immunological changes implicated in the development of immunosenescence include cumulative alterations in B and T cell populations, increased levels of memory cells producing Th1 and 2 cytokines and T cell dysfunction due to thymic degeneration [73,74]. It has been suggested that thymic degeneration with age may lead to an accumulation of T cells that are reactive to neo(auto)antigens [74] and therefore increase the likelihood of a breakdown in self tolerance, as well as susceptibility to disease-triggering infections.

Though the total T cell number does not appear to be significantly decreased with age, there is an alteration of T-cell receptor (TCR) signalling, as well as TCR re-expression, namely due to decreased CD28 expression [75]. CD28 is a co-stimulatory receptor involved in antigen-mediated T cell activation, proliferation and survival [76-78]. Virtually all naive T cells in the umbilical cord are CD28+ [79]. So called CD28 null cells (CD28-), which do not express the CD28 receptor, are functionally active oligoclonal lymphocytes that lack proliferative capacity, have reduced antigen receptor diversity and defective antigen induced proliferation hence are senescent cells [76,78]. It has emerged that the accumulation of CD28- cells occurs consistently with age, and CD28- T cells are predictive of immune system incompetence in the elderly [76,78]. By the age of 80, 10-15% of peripheral blood CD4+ and 50-60% CD8+ T cells lack CD28 [78]. Increased CD28- cell accumulation is associated with a reduced response to pathogens and vaccines [76,78], which is reflected in the low response to the influenza virus in the elderly [80]. In addition, CD28- cells have been found in patients with chronic inflammatory syndromes of all age groups, indicating a premature senescence of lymphocytes due to persistent immune activation [76,78]. Repeated stimulation of purified CD28+ cells with a specific antibody to the T cell receptor (TCR)/CD3 complex, results in decreased CD28 expression, and an increased proportion of CD28- cells [76]. Increased stimulation with IL-15 and TNF-α has also been shown to augment the loss of CD28 [78]. The loss of CD28 is therefore characteristic of both ageing, as well as chronic inflammatory disease. CD8+CD28- cells are more common in patients with autoimmune disease, and their increased effector functions may enhance autoimmune disease and inflammatory responses [78]. CD8+CD28- T cells accumulate due to normal ageing, but also reflect chronic infection with viruses such as Epstein-Barr virus, human immunodeficiency virus, and human cytomegalovirus [78,81]. The loss of immunological tolerance and increased susceptibility to infections is interesting in the case of PBC, as numerous infectious agents have been implicated in the immunopathogenesis of PBC, by mechanisms such as molecular mimicry and immunological cross-reactivity [47,58,62,82-91].

The changes noted above have not been specifically linked to PBC, but do give a general view of immunological alterations which may contribute to autoimmunity as we progress in age (Table 1). However, several phenotypic and functional changes have been noted in the CD4 and CD8 population of PBC patients [92]. As mentioned, PBC is histologically characterised by a dense mononuclear cell infiltrate, populated largely by CD4 and CD8 T lymphocytes [1-3,34]. Additionally, higher numbers of CD4 and CD8 T lymphocytes are found in the liver than in the peripheral circulation of PBC patients [92]. This has led to the suggestion that CD8+ cells may mediate BEC damage [92]. Bernuzzi and colleagues examined CD8 T regulatory (Treg) cells in PBC patients, and found several phenotypic and functional alternations [92]. Phenotypically, CD8 Treg from PBC patients had increased CD127 and reduced CD39 expression [92]. As CD127 is a marker of effector function whilst CD39 is indicative of normal Treg function, this abnormal phenotype may be indicative of a failure of competent Treg functioning [92]. There was no significant difference noted in the number of CD8+CD28- cells in PBC patients versus controls, however, CD8+CD28- cells from PBC patients failed to exhibit a regulatory immune response after incubation with IL-10 [92]. Interestingly, CD4+CD25+ function was not altered in PBC, which differs from other autoimmune disease [92]. These data indicate that alterations in CD8+Treg and failure of regulatory mechanisms are a feature of PBC.

### Telomere and Telomerase activity is altered in ageing and in PBC

Telomeres are located at the ends of chromosomes, are composed of repeated DNA sequences and ensure that each DNA replication cycle has been completed [93]. With each division, sequences of the telomeres are lost, forming the basis for the hypothesis that telomere shortening occurs with age. Moreover, the loss of telomere sequences can be accelerated through oxidative stress and inflammation [93]. Short or damaged telomeres may induce DNA breaks, initiate cell senescence, or induce apoptosis [93]. Senescence occurs at a particular telomere length, at which point the cell passes the so called 'mortality checkpoint 1', which is under control of p53 and Rb genes [93]. Further loss of telomere length and passage through mortality checkpoint 2, will induce apoptosis [93]. These events could in turn result in a loss of immunological tolerance [93] and it has been observed that shortened telomere length is a feature of many autoimmune diseases [94-103] including PBC [104]. Of note, increased disease activity has been correlated with increased telomere shortening in systemic lupus erythematosus (SLE) [103].

Telomerase is an enzyme which prevents telomere shortening, and may also increase telomere length [93]. Telomerase activity varies among autoimmune diseases [93], for example, there is increased telomerase activity in SLE [105], RA [106], juvenile idiopathic arthritis [107] and adult onset still disease [108], whilst telomerase activity is unchanged in Wegener's granulomatosis [109], and even reduced in systemic sclerosis [106].

Shortened telomere length with associated cellular senescence has been well demonstrated in a study by Sasaki and colleagues [104]. In that study, the BEC telomere length of 13 PBC patients, as well as 13 control subjects (consisting of liver samples from healthy patients and chronic viral hepatitis), was examined using quantitative fluorescent in situ hybridisation [104]. In addition, immunohistochemical studies for the senescence markers p16INK4a and p21WAF/Cip1, as well as for DNA damage, were performed [104]. A significant decrease in telomere length was noted in damaged BEC from PBC patients, in comparison to histologically normal BEC in PBC patients and controls [104]. Immunohistochemistry demonstrated γH2AX-DNA damaged foci in BEC of PBC patients, with cellular senescence confirmed by positive p16INK4a and p21WAF/Cip1 staining [104]. These changes were not observed in healthy or pathological controls, and suggest that telomere shortening with associated DNA damage and cellular senescence is a unique feature of damaged BEC in PBC [104]. It should be noted that it was not possible to correlate telomere length with age, due to the small cohort size and telomerase activity was not examined. Further confirmatory studies using a larger cohort, as well as an analysis of telomerase activity in the BEC of PBC patients is warranted.

### Senescence and autophagy

Senescence is a delayed stress response, which is induced by factors such as telomere shortening, oxidative stress, and DNA damage [110,111]. Senescence may also be induced by ageing cells, as a mechanism for limiting the depletion of cells in ageing tissue [110]. Several studies have demonstrated that oxidative stress is an inducer of cell senescence in the BEC of PBC patients [104,112-114]. Sasaki et al [112] notes that cellular senescence in early-stage PBC was associated with an infiltration of myeloperoxidase-positive inflammatory cells, indicating oxidative stress. In that study, BEC showed increased p21WAF/Cip1 staining, and decreased Ki67 (a marker of proliferating cells) staining [112]. Another study by the same group examined the expression of the Bmi1 gene (which suppresses p16INK4a activity) in liver samples from 18 PBC cases, 19 pathological controls, and 16 healthy livers [114]. Bmi1 was significantly reduced in damaged BEC of PBC patients, compared to strong expression in the nuclei of control groups [114]. In addition, cultured mouse BEC subjected to oxidative stress from H2O2 exposure had significantly decreased Bmi1 expression, and increased p16INK4a activity [114].

Senescence is of interest in the development of PBC for several reasons. Firstly, senescent cells promote an environment which is pro-inflammatory and pro-fibrotic through secretion of cytokines (such as IL-1 and IL-6) and chemokines (IL-8 and MCP-1) [46,111,115-118]. Secondly, the process of autophagy, or "self eating", is involved in the maintenance of senescence, and may provide a route for BEC damage, as well as exposure of autoantigens into an established inflammatory environment [111,117,119]. Autophagy is a process whereby intracellular proteins and organelles are degraded to suppress damage and maintain metabolism of senescent cells [120-123]. An immunohistochemical study by Sasaki and colleagues investigated whether autophagy was associated with senescence in PBC [119]. Liver samples from 37 PBC and 75 controls were stained for autophagy markers LC3, cathepsin-D, and LAMP-1, as well as for senescence markers p16INK4a and p21WAF/Cip1 [119]. LC3 expression was present in 90.5% of inflamed and 27.5% non-inflamed bile ducts of PBC patients [119]. LC3 also co-localised with cathepsin-D, LAMP-1, p16INK4a and p21WAF/Cip1 [119]. These findings were not observed in the control group. Cultured BEC subjected to oxidative stress (with hydrogen peroxide), DNA damage (with Etoposide), and serum deprivation, induced cellular senescence [119]. Inhibiting autophagy with 3-methyladenine also reduced stress induced senescence, including a reduction of senescence associated phenotype markers CCL2 and CX3CL1 [119]. Although senescence and autophagy may play a role in BEC damage and exposure of cryptic epitopes, the competitive process of apoptosis may also be implicated in the pathogenesis of PBC.

### Apoptosis: potential source of autoantigens

Alterations in the apoptotic process in PBC may provide a source for BEC autoantigens, as well as limiting the immune systems ability to clear infections. Cell shrinkage, chromatin condensation, DNA cleavage, and packaging of cellular material into apoptotic bodies called blebs are all features of apoptotic cells [124,125]. The apoptotic process may be initiated as an alternative to senescence and autophagy, by triggers such as cytotoxic or oxidative stress, cytokine deprivation, or genomic damage [124,125]. Apoptotic cells release "find me" signals such as soluble lysophosphatidylcholine, and "eat me" signals such as phosphatidylserine [125,126]. These signals are recognised by phagocytes, which bind the apoptotic cell [125,126]. Phagocytes which bind the apoptotic cell release anti-inflammatory cytokines such as IL-10, and also induce a reduction of inflammatory cytokines such as IL-1β, TNF-α, and IL-12 [125,127,128]. Deficiencies in this process may result in the release of cellular material, which could be presented by antigen presenting cells to autoreactive B and T cells, thereby provoking inflammation and autoantibody formation [124,125,129-131]. In addition, the apoptotic blebs appearing on the surface of apoptotic cells can act as a source of autoantigens [132-138].

Apoptosis has been shown to play a role in several autoimmune diseases, such as SLE [129-131,139-142] and PBC [111,132,134-136,142-144]. However, several unique characteristics are found in the apoptotic processes of PBC versus SLE. Lorenz and colleagues note an increased number of apoptotic peripheral lymphocytes from SLE patients *in vitro*, which relates to an in vivo increase in these cells activation [142]. Increased numbers of apoptotic cells were found in the skin of patients with cutaneous lupus, after exposure to UV light [141], and remnants of apoptotic cells were found in the germinal centres of lymph nodes in SLE patients [139]. Additionally, tingible body macrophages, which clear apoptotic cells in healthy individuals, were noted to have abnormal morphology in SLE patients [139]. It was demonstrated by Koga et al [143] that apoptosis is a feature of BEC in PBC patients. That study involved 35 PBC patients, 16 normal controls, and 17 chronic hepatitis C patients [143]. *In situ *nick-end labelling showed increased DNA fragmentation in BEC of PBC patients, and immunohistochemical analysis of Bcl-2 showed that it was more frequently expressed in the BEC and hepatocytes of PBC patients [143]. Ten of the PBC patients underwent a second liver biopsy after treatment with urseodeoxycholic acid, the treatment of choice for PBC, and it was found that there was decreased Bcl-2 expression in the BEC, and decreased DNA fragmentation [143]. Kuroki et al [144] also indicates that the Fas pathway of apoptosis may be involved in PBC, as BEC showed increased Fas (CD95) on their cell membranes.

Studies into apoptosic pathways in PBC have also provided interesting data in regards to why AMA are so highly specific for PBC. Odin and colleagues [136] note that the recognition of PDC-E2 still occurred in apoptotic BEC. The PDC-E2 was intact, as it had not been cleaved by caspases [136] and autoantibody recognition of PDC-E2 was also maintained due to a lack of glutathionylation [136]. Lleo et al [135] found that unmodified PDC-E2 localised into the apoptotic blebs in human intrahepatic BEC, called apotopes [135]. Sera from AMA positive individuals reacted with PDC-E2 on apoptotic BEC, demonstrating an accessibility of PDC-E2 to the immune system [135]. The same group of investigators have also demonstrated that BEC apotopes in the presence of macrophages from PBC patients, as well as AMA, induced the production of inflammatory cytokines [134]. Finally, it has also been demonstrated that the phagocytosis of apoptotic BEC by BEC provides these cells with a source of mitochondrial self-peptide [132].

The studies mentioned so far in this review demonstrate that immunological changes which occur with age may promote an increased susceptibility for PBC development. Although these changes occur in normal ageing, it appears that they may be altered, or even accelerated in individuals with a genetic predisposition, or who are exposed to other triggers implicated in PBC development. Oxidative damage and decreased immunological tolerance open the door to the suggestion that infectious agents may also be involved in PBC development. Several features of the infectious model underlying PBC development appear to be unique to females, which may explain the female predominance of PBC.

### Recurrent Urinary Tract Infections and *E. coli*

Several large epidemiological studies have consistently indicated that recurrent UTIs are a risk factor for PBC development [61,63,64,145]. The association between UTI and PBC was first made by investigators at the Royal Free Hospital in London, who found an increased rate of bacteriuria in female PBC patients, compared to controls [89,146]. One study reported recurrent UTI in 19% of PBC patients, compared to 5-6% of controls [89]. 57% of patients also reported at least one bacteriuric episode and more than two episodes were reported in 26% of PBC patients [89]. Parikh-Patel and colleagues demonstrated a positive association between PBC and recurrent UTI [63]. A large and comprehensive study by Gershwin et al [145] demonstrated that 59% of 1032 PBC patients reported UTI. A relatively recent study by Prince and co-workers [64] also demonstrated an association of PBC with UTI. That study involved one group of 318 PBC patients from a geographically defined area, and another involving 2258 patients recruited from a PBC support group [64]. Multivariate analysis showed an association between UTI and PBC in PBC patients from both groups, but not in a cohort of 3936 demographically matched controls [64].

The link between UTI and PBC has been limited, as it is unclear as to whether recurrent UTI preceded PBC diagnosis, or is sequelae of concomitant autoimmune disease, such as Sjögren's/Sicca syndrome. It could be argued that changes in mucosal immunity which occur in these conditions, increases the risk of developing UTI. However, a recent study by Varyani and colleagues [147] has demonstrated that UTI preceded the diagnosis of PBC in a large cohort of PBC patients. Using the General Practice Research Database, the investigators identified 800 PBC patients, 7991 matched controls from the general population and 12 137 chronic liver disease control patients [147]. UTI had been diagnosed within one year prior to PBC diagnosis in 29% of PBC patients, compared to 22% of matched controls and 17% of chronic liver disease controls [147]. Within a five year period prior to diagnosis, 19% of PBC patients had been diagnosed with UTI, compared to 14% of matched controls and 11% chronic liver disease controls [147]. Of note, the mean age of PBC diagnosis was 63 years [147]. Whether PBC patients are more prone to UTI, or whether the increased incidence of UTI in PBC patients is due to natural ageing is unknown, as the incidence of asymptomatic and symptomatic bacteriuria increases with age [148]. *Escherichia coli *(*E. coli*) is the most commonly identified organism, being isolated in 85.7% of male and female UTI patients aged 15-65 years, and in 74% of patients over 65 years [148].

*E. coli *has been identified as an organism of interest, due to its frequency as a causative organism in UTI and being implicated in up to 70% of PBC patients with recurrent UTI [89]. Early studies have been focused particularly on rough forms of *E. coli *and their relation to PBC [86,89,149]. We and others have investigated the role of microbial/self molecular mimicry and immunological cross-reactivity as a mechanism responsible for the induction of liver autoimmunity, including that seen in patients with PBC [54,91,150-160]. Support for the molecular mimicry hypothesis in PBC has been found on the experimental level, where sera from PBC patients react with both *E. coli *and human PDC-E2 [161]. Interestingly, sera from 52% of patients with PBC reacted with PDC-E2 in one cohort [162]. Several studies have noted a cross reactivity between antibodies in PBC patients with amino acid sequences inside the ATP-dependant Clp protease of *E. coli *[86,163,164]. Similar lipoyl domain sequences are also found between *E. coli *and human PDC-E2 and these sequences are essential for T cell epitope recognition of PDC-E2 in humans and *E. coli *[83,91]. The same study found a strong association between this reactivity and a history of recurrent UTI [83]. Shimoda et al provided data clearly demonstrating that *E.coli *and human PDC-E2 homologues are targets of cross-reactive responses at the CD4 T-cell level [91,165,166]. Of note, these epidemiological and experimental studies demonstrate a potential link between two pathologies with a high female preponderance.

In addition to *E. coli*, *Lactobacillus delbrueckii *(*L. delbrueckii*) is emerging as an important organism in the link between recurrent UTI and PBC as it may be both synergistically and directly pathogenic. *L. delbrueckii *is a component of normal vaginal flora, and alterations in this flora may lead to recurrent vaginitis, which in turn may lead to colonisation by bacteria such as *E. coli *and hence recurrent UTIs [167]. Vaginitis and vaginal infection has also been noted as being more prevalent in PBC patients [63,145]. It is therefore plausible that *L. delbrueckii *contributes to the development of recurrent UTI, by creating an environment which is prone to colonisation by bacteria such as *E. coli*. In addition, recent studies have also demonstrated that *L. delbrueckii *can be a directly pathogenic organism of UTI in elderly women [168,169].

Lactobacilli have also been linked to PBC development in a 39 year old female who received several Lactobacilli vaccinations for recurrent vaginitis [25]. AMA from this patient reacted against human PDC-E2, and was also cross reactive with beta-galactosidase of *Lactobacillus delbrueckii *[25]. It has been previously identified that *L. delbrueckii *contains epitopic regions which mimic PDC-E2 [167]. Hence, in women, *L. delbrueckii *may be implicated directly, through molecular mimicry, and indirectly, by predisposing to recurrent UTI in the pathogenesis of PBC. Several other bacteria, viruses, and parasites are being investigated as potential triggers of PBC via molecular mimicry and cross reactivity, but one in particular, *Novosphingobium aromaticivorans*, may also be relevant to female preponderance.

### *Novosphingobium aromaticivorans*, oestrogen, and reproduction

*N. aromaticivorans *is a gram negative bacteria found universally in air, soil, and water [170]. Of relevance to PBC this bacterium contains two proteins which share amino acid sequences with the immunodominant epitope of PDC-E2 [170]. *N. aromaticivorans *has also been found to induce PBC-specific anti-mitochondrial antibodies in immunised mice that develop liver pathology with histological characteristics similar to that seen in PBC [171].

Several studies have indirectly alluded to oestrogen deficiency as a potential risk factor for PBC development [61,64,145,172,173] and *N. aromaticivorans *has been shown to metabolize xenobiotics and oestrogen [174]

The intracellular pathways involving the stimulatory affects of oestrogen on cholangiocyte proliferation has been investigated by Alvaro and colleagues [174-176]. Animal models have shown that oestrogens and other growth factors induce cholangiocyte proliferation via activation of the Src/Shc/ERK molecular signalling cascade [176]. Alvaro and colleagues suggest that there is a synergistic role between oestrogens and growth factors in cholangiocyte proliferation [176]. Antagonists to oestrogen receptors (OR) also inhibit cholangiocyte proliferation and reduce expression of ERK/Shc [176]. Exogenous oestrogen administration with 17-β-oestradiol increases Src/Shc/ERK protein expression as well as cholangiocyte proliferation *in vitro *[176]. These results demonstrate that oestrogens likely play a role in cholangiocyte proliferation [174-176].

The same group of investigators also demonstrated a role for oestrogen in the progression of PBC [174]. Liver biopsies from PBC patients and controls (normal liver, primary sclerosing cholangitis and alcoholic cirrhosis) were immunohistochemically examined for oestrogen receptor alpha (OR-α), oestrogen receptor beta (OR-β), cytokeratin 19, proliferating cellular nuclear antigen (PCNA), and Fas by terminal deoxynucleotide transferase end labelling (TUNEL) [174]. OR-α and OR-β were only found in PBC samples [174] and OR-β was present in all histological PBC stages in 50-65% of PBC patients. OR-α expression varied based on histological stages, with OR-α being expressed in 1% of those in stage 1, 12% in stage 3, and absent in stage 4 [174]. This correlated with PCNA expression and maximal ductopaenia [174]. OR-α was significantly decreased in PBC compared to that seen in pathological controls [174]. These findings suggest that OR-α expression is reduced in advanced PBC, which favours a progression towards ductopaenia [2,174]. Interestingly, an early study indicated that exogenous oestrogen administration improved liver biochemistry in PBC patients [177]. Those researchers noted a fall in serum enzymes at mid-menstrual cycle in untreated PBC patients [177]. Five PBC patients were administered ethinyl oestradiol: 1 premenopausal, 1 post-menopausal, and 3 post-oophorectomy [177]. Aspartate transaminase decreased by greater than 50% after 2 weeks of treatment in 4 of the 5 patients, γ-GT decreased by 50% in all 5 patients, and alkaline phosphatase decreased by 30% [177]. Repeated courses of oestradiol administration produced similar results [177]. Although this study was limited in terms of controls and cohort size, it is interesting given the more recent findings indicating the protective effects of oestrogen in PBC, as well as a decrease in hormone replacement therapy reported in some studies [64,145].

Apart from oestrogen, several characteristics in the obstetric histories of women with PBC are worth noting. Parikh-Patel and colleagues have noted an increased incidence of sexually transmitted infections in female PBC patients *versus *controls (18.5% versus 10.4%), as well as an increase in vaginal infection (63.4% versus 35.2%)[173]. Epidemiological studies have also indicated that PBC patients tend to be younger at the time of their first pregnancy, as well as having an increased incidence of pregnancy termination and extrauterine pregnancy [61,63,64,145]. Investigation into the reproductive characteristics of PBC patients therefore warrants further study.

### Viruses and ageing in PBC

Age related changes in the ability of antigen-specific cellular immune responses to clear viruses have been noted. This has led to the assumption that ageing as a risk for autoimmunity may be due to the inability to erase viral-triggered autoimmune responses [74,75]. Whether this is possible in the case of PBC is still unknown. Geographical, clinical, and experimental evidence has led to the suggestion that a viral cause may underline the development of PBC in some individuals [178,179]. Clusters of PBC have been noted among individuals who emigrate from areas with low PBC rates to areas with high PBC rates, and unrelated individuals living in the same household have developed the disease [178]. As well, recurrence of PBC after liver transplantation appears to occur earlier, and with a greater severity in patients treated with tacrolimus, and the opposite is observed with cyclosporine administration [178,180]. The group led by Mason and colleagues have found evidence of viral particles in the biliary epithelial cells of PBC patients, and antibody reactivity to retroviruses has been found in the sera of PBC patients [181]. This reactivity was found to be specific to viral proteins when reactivity to AMA was blocked out [182]. Genetic material of a human betaretrovirus (HBRV) (with 95% homology to mouse mammary tumour virus, or MMTV) has been detected in the lymph nodes of ~75% of PBC patients in a cohort of PBC patients [183]. Further analysis by immunohistochemistry also demonstrated HBRV material in the lymph nodes of PBC patients, which was not observed in controls [183]. However, lower detection rates were observed when hepatocytes were analyzed (only ~30%), as well as in other studies utilising a PCR approach [183,184]. Selmi et al [184] were unable to detect reactivity between PBC sera and a mouse MMTV, and no immunohistochemical or molecular evidence for MMTV was found in liver specimens. In contrast to this, Johal and colleagues found MMTV sequences in healthy liver tissues, in addition to liver tissue from a variety of liver diseases including PBC [185]. Selmi notes that reactivity between PBC sera and some viral proteins may be due to the presence of mitochondrial antigens in the viral preparations derived from murine MMTV [184]. However, the differing rates of viral detection from one study to the next may be due to differing methodological approaches [178]. Mason [178] highlights that studies by his group utilised a RT-PCR approach, which is more sensitive for detecting retroviral material than PCR which was used in the study by Selmi [184]. As well, other studies have not examined lymph node material from PBC patients, despite these tissues being the major reservoir for HBRV [178,183]. Lymph nodes co-cultured with normal cholangiocytes were shown to induce the expression of AMA-antigens on the cholangiocyte cell membrane [186]. Another study has demonstrated that HBRV infected cells expressed the PDC-E2 phenotype, and *in vitro *cell cultures were induced to express PDC-E2 when exposed to HBRV [187]. Of interest, a randomized controlled trial with anti-retroviral therapy is planned to treat patients with PBC who are unresponsive to UDCA [178,188,189], although similar studies [190] have failed to reach non-histological endpoints utilized in clinical trials in PBC [191].

A viral role in the pathogenesis of PBC is still under debate [191]. Several factors may account for this, such as the fact that only a small number of studies have been performed, as well as the conflicting results generated by different groups [191]. As well, differences in experimental approach and design, as well as authors interpretation of the results, may also account for the inconclusive status of viruses in the pathogenesis of PBC [178]. Further studies are needed to provide a conclusion in either direction.

### Concluding Remarks

Primary biliary cirrhosis is an autoimmune disease with a striking predominance in middle-aged females. Various genetic characteristics and environmental factors have been implicated in the disease development and progression. Factors, such as recurrent UTI and oestrogen signalling provide insight as to why females are more prone to PBC. Studies characterising the decline of the immune system with age, have provided evidence as to why presentation with PBC often occurs in middle age. It is likely that genetic predisposition combined with environmental triggers act in a multiple hit model. These factors may also contribute to the decline of an already ageing immune system, through recurrent infections and inflammation, as well as oxidative damage. The immune system may then reach a critical stage where tolerance to self is lost. Increased cellular stresses via environmental or infectious triggers, may also contribute towards a propensity of apoptosis in the BEC, which allows for autoepitope exposure to the immune system.

## Abbreviations

AMA: antimitochondrial antibodies; ANA: antinuclear antibodies; BEC: biliary epithelial cells; E.coli: Escherichia coli; FDR: first degree relatives; OR: oestrogen receptor; PBC: primary biliary cirrhosis; PDC: pyruvate dehydrogenase complex; RA: rheumatoid arthritis; SLE: systemic lupus erythematosus; TCR: T-cell receptor; UTI: urinary tract infections.

## Competing interests

The authors declare that they have no competing interests.

## Authors' contributions

DSS wrote the first draft; Subsequent drafts were written by DSS, EIR and DPB, who had the overall supervision of the review processing; all authors edited the paper and approved its final version.
